# Gastric Cancer with Radiographically Occult Metastatic Disease: Biology, Challenges, and Diagnostic Approaches

**DOI:** 10.3390/cancers12030592

**Published:** 2020-03-05

**Authors:** Aravind Sanjeevaiah, Haeseong Park, Benjamin Fangman, Matthew Porembka

**Affiliations:** 1 Department of Medicine, University of Texas at Southwestern, Dallas, TX 75390, USA; BENJAMIN.FANGMAN@phhs.org; 2 Department of Medicine, Washington University School of Medicine in St. Louis, St. Louis, MO 63110, USA; haeseongpark@wustl.edu; 3 Department of Surgery, University of Texas at Southwestern, Dallas, TX 75390, USA; Matthew.Porembka@UTSouthwestern.edu

**Keywords:** gastric cancer, peritoneal disease, ctDNA, staging laparoscopy

## Abstract

Gastric adenocarcinoma is an aggressive cancer that demonstrates heterogeneous biology depending on patient ethnicity, tumor location, tumor type, and genetic profile. It remains the third leading cause of cancer deaths worldwide and was estimated to result in 782,000 deaths in 2018. Challenges exist in accurately assessing the disease burden, as available radiological staging often underestimates metastatic disease. This diagnostic handicap, along with the poor understanding of the heterogeneous biology of gastric cancer, has hindered the development of effective therapeutic solutions and thus halted improvement in patient outcomes over the last few decades. The management of occult peritoneal disease is complicated, as most patients are understaged by standard imaging studies and therefore thought to have local diseases. In this article, we systematically review recent literature on the limitations that are associated with standard radiographic staging, discuss recent molecular biology advances to better identify and diagnose occult peritoneal disease, and propose possible management strategies to approach this complicated clinical problem.

## 1. Background and Radiology Limitations

Gastric cancer represents a significant global burden, most notable in Asian countries. In the United States, 27,500 new gastric cancers and 11,100 gastric cancer-related deaths were expected in 2019 [1]. Globally, 782,000 deaths were estimated in 2018 [2]. Gastric cancers are heterogeneous cancers in regards to location (proximal vs distal gastric cancers), histology (intestinal vs diffuse), and race (different clinical behavior and stage specific survival likely due to different biology). Distinct clinical entities have been identified by using whole genome sequencing and molecular analysis [3,4]. Though various molecular and genetic groups have been defined and described (See Table 1 and Table 2), meaningful clinical translation and adaptation into clinical practice has yet to take place.

Historically, the heterogeneity associated with gastric cancer has not been well-recognized, thus leading to clinical trials that lacked specificity and treated all gastric cancers, and often esophageal cancers, as a combined clinical entity. Consequently, there has been only an incremental improvement in clinical outcomes over the last several decades. The Intergroup 0116 study showed an improvement in three-year survival from 41% to 50% with adjuvant chemoradiation therapy compared to surgery alone [5]; this regimen is currently still utilized in the setting of poor surgical quality control related to positive margins or inadequate lymph node dissection. Adjuvant chemotherapy is recommended for patients who receive upfront surgery without neoadjuvant chemotherapy, and this practice is supported by two high-quality Asian studies that demonstrated an improved overall survival among patients receiving adjuvant chemotherapy [6,7]. The UK Medical Research Council Adjuvant Gastric Infusional Chemotherapy (MAGIC) trial established peri-operative therapy as the standard of care [8]. Not only did this trial demonstrate significant improvement in five-year overall survival from 23% to 36% with the triplet chemotherapy of Epirubicin, cisplatin and 5 Fluorouracil compared to surgery alone, it also demonstrated that peri-operative therapy was better tolerated than chemotherapy that was administered in the adjuvant setting. After a decade of negligible progress, the AIO/FLOT4 trial that used the docetaxel-based triplet FLOT (5-FU, folinic acid, oxaliplatin, docetaxel) chemotherapy demonstrated a 45% five-year overall survival compared to 36% in the ECF/ECX control group [9]. Though the use of FLOT was associated with increased treatment response and complete pathologic response rates, surgical resection remains the only curative option that is available for patients with localized gastric cancer.

Accurate staging prior to surgery is critical in the management of localized gastric cancer for appropriate treatment planning and accurate prognostication. Unlike Asian countries with well-developed screening endoscopy programs [10,11], the majority of Western patients present with advanced diseases. Even in patients thought to have localized diseases, the suboptimal survival of Western cohorts compared to those of the East is likely related to differences in surgical quality control and inadequate staging. Occult peritoneal disease is a frequent cause of inadequate staging in gastric cancer. Peritoneal disease is difficult to detect based on current imaging tests, and a high index of clinical suspicion is required to accurately diagnose and treat these patients.

Patient- and tumor-related factors are associated with an increased risk of occult peritoneal disease. Diffuse histology, along with higher T and N stage diseases are associated with early peritoneal spread in patients with gastric cancer [12]. Despite the reasonable T and N staging abilities of computer tomography (CT), magnetic resonance imaging(MRI), and positron emission tomography (PET), these modalities are highly limited in their ability to identify low-volume peritoneal disease [13]. Computer tomography is the most widely used modality for staging in gastric cancer, but it is ineffective in detecting low volume peritoneal disease. Between 20 and 30 percent of patients who have T2 or deeper tumors on Endoscopic Ultrasound (EUS) have peritoneal metastases despite having a negative CT scan [14,15,16]. The PET/CT scan has been studied in the context of the staging of gastroesophageal cancer and, other than in gastroesophageal junction tumors, it has no role in the assessment of occult peritoneal disease due to the limited resolution of PET scans [17]. This is especially true of diffuse-type cancers that have lower baseline SUV compared to intestinal-type cancers [18].

Given the limitations of radiographic staging, staging laparoscopy has become an important adjunct to accurately stage patients and prevent unnecessary invasive operations. Currently, staging laparoscopy is recommended for T1b or higher gastric cancers [19]. Reported positive laparoscopy rates are highly variable (13–63%), owing to variable study inclusion criteria [20,21,22,23,24,25,26,27,28,29,30,31,32,33,34,35]. The majority of the literature regarding laparoscopy has been published by Asian centers [20,21,24,29,31,32,33,36,37] where the indication for staging laparoscopy is often the presence of locally advanced diseases, defined as nodal involvement or T3/T4 disease without evidence of metastasis [10,11,20,21,29,30,31,32,36,37]. The yield of laparoscopy is related to the disease stage. In studies limited to patients with locally advanced diseases, metastases were frequently identified (29–63%) [20,21,29,30,31,32,36,37]. As expected, when patients with earlier stage diseases undergo routine laparoscopy, the yield decreases but continues to have a considerable clinical impact (17–41%) [12,23,28,34,35,38,39,40]. The yield of staging laparoscopy appears to be higher in distil gastric cancers compared to Gastroesophageal (GE) junction tumors and diffuse gastric cancers compared to intestinal-type cancers [10,41]

In addition to white light laparoscopy, peritoneal lavage should be obtained at the time of diagnostic laparoscopy for cytological analysis. Even in the absence of overt peritoneal spread, the presence of malignant cells in the peritoneal cytology is a poor prognostic sign [42,43] and is treated a clinical metastatic disease. Traditionally, the conventional cytological evaluations of peritoneal fluid by using Papanicolaou or hematoxylin and eosin stains has been employed, but this method has a low sensitivity and a poor negative predictive value. Newer techniques like immunoassays, immunohistochemistry (IHC), and reverse transcriptase-polymerase chain reaction (RT-PCR) have been developed for improved sensitivity in detecting intraperitoneal-free cancer cells, and RT-PCR appears to have the highest sensitivity among the newer techniques [43]. Patients with positive peritoneal cytology without gross peritoneal disease who undergo gastrectomy recur and have a prognosis similar to patients with M1 diseases; therefore, surgery is not routinely offered without evidence of favorable biology. Recently, two retrospective studies examining patients with limited peritoneal disease or positive cytology demonstrated that treatment with cytoreductive surgery and hyperthermic intraperitoneal therapy in conjugation with perioperative chemotherapy was associated with improved survival [44,45]. In one study an exceptionally high five-year overall survival of 46.8% was demonstrated for patients with a peritoneal cancer index (PCI) of less than 6. A prospective multicenter randomized clinical trial (PERISCOPE II) is currently underway to investigate the utility of radical gastrectomy with cytoreductive surgery and hyperthermic intraperitoneal chemotherapy in patients with limited peritoneal disease and/or positive peritoneal cytology who did not progress after initial systemic chemotherapy [46].

## 2. Biology

Substantial literature on the molecular biology of gastric cancer is now available thanks to the recent revolution in high-throughput technologies. Building on the available literature and through stellar work on high-throughput techniques, two groups have recently proposed the molecular classification of gastric cancer [3,4].This has revolutionized our understanding of gastric cancer and will undoubtedly result in better clinical care in the future. Table 1 and Table 2 summarize the characteristics of molecular subgroups that were proposed by the ASRG and TCGA groups.

Data on the molecular biology of gastric cancers that present with low volume peritoneal disease, however, are sparse. It is possible these cancers merely represent gastric cancers that are early in the disease process and will ultimately declare themselves with larger peritoneal involvement and may also metastasize to distant organs. It is also possible that the biology of the subset of cancers that includes low volume peritoneal disease, especially cancers with diffuse histology, is different. This might particularly be true for those metastatic gastric cancer patients with only peritoneal metastasis whose affected cohorts present with profound weight loss and sarcopenia and ultimately succumb to the disease without any non-peritoneal metastatic involvement. While there are no specific data on the molecular characteristics of this specific subgroup of patients, it is possible to get an insight through an analysis of a subset of patients with these characteristics in the available literature.

The most intriguing data came from the molecular analysis of gastric cancers by the Asian Cancer Research Group, who identified a subgroup of gastric cancer, microsatellite stable/epithelial-to-mesenchymal transition (MSS/EMT) that was highly associated with peritoneal disease. This subset accounted for accounted for 15% of the cohort, was more likely to result in peritoneal metastasis (64% vs 23 %), and was unlikely to develop liver metastasis (4.6%). They originated mostly in the gastric antrum (37%) or the body (46%), had diffuse-type histology (80%), and presented at advanced stages (III/IV 80%). The patients in this group were 10 years younger than the other groups (median age: 53 years). This subtype had the worst overall prognosis and recurrence-free survival. Though 80% of MSS/EMT tumors were diffuse, only 27% of all diffuse gastric tumors were captured in this subgroup. Given the high risk for this subgroup to present with peritoneal disease, they are at the greatest risk for understaging secondary to the presence of radiographically occult peritoneal disease [3,4]. MSS/EMT tumors appear to comprise the subgroup of diffuse gastric cancers with the poorest prognosis.

While the genomically stable (GS) subgroup of TCGA appears to be enriched for distal and diffuse gastric cancers, the GS and MSS/EMT subtypes do not appear to be identical [47]. TCGA and ASRG used different techniques for molecular classification. Nonetheless, the TCGA data provide the molecular basis for the poor survival of diffuse gastric cancers.

## 3. Management

The management of radiographically occult peritoneal disease, either diagnosed by cytology or laparoscopy, is challenging. Unlike visceral metastasis, the ability to assess treatment response is impossible by conventional cross-sectional scans, thus leading to continuation of potentially ineffective therapy until clinical progression (as manifested by the development of malignant ascites, symptomatic obstruction, weight loss, and the deterioration of nutritional and performance status). Novel and reliable methods to assess response are needed effectively guide clinical care.

Patients with peritoneal disease have shorter survival despite palliative chemotherapy. However, it is unclear whether this shorter survival is due to resistance to frontline chemotherapy or due to the poor performance status that is often secondary to poor nutritional status and that often accompanies peritoneal metastasis. Diffuse gastric cancers that are associated with early peritoneal spread have been long recognized to respond less favorably to chemotherapy in adjuvant trails. In the seminal intergroup study, diffuse gastric cancers had no benefit with adjuvant chemoradiation therapy [5]. The subgroup analysis of the ARTIST trial showed that diffuse gastric cancer derived lesser benefits with chemoradiation therapy compared to the intestinal subgroup [48]. Pattison et al. showed early relapses after adjuvant chemotherapy in an Australian population, thus suggesting primary chemoresistance in diffuse gastric cancer [49]. Increased RhoA activity in diffuse gastric cancer has been hypothesized to promote cancer stem cell-like phenotypes and chemoresistance, and RhoA inhibition might reverse chemoresistance [50]. A recent meta-analysis that included nearly 61,000 patients showed that patients with a diffuse histology had a worse prognosis than those with an intestinal-type histology (HR: 1.23; 95% CI: 1.17–1.29; *p* < 0.0001) in the loco-regional and advanced stages (HR:1.21; 95% IC: 1.12–1.30, *p* < 0.0001 and HR: 1.25; 95% IC: 1.046–1.50; *p* = 0.014, respectively), with or without neoadjuvant treatment [51]. While the Phase III FLOT4 study showed that a FLOT regimen was effective in patients with signet ring cells (HR: 0.74 versus 0.79 in the intestinal subgroup), thus confirming the utility of this regimen, the earlier phase II FLOT4 study reported overall pathological responses in intestinal histology of 23% and 10% in the FLOT and ECF/X arms, respectively, versus just 3% in both groups in the case of diffuse gastric cancers [9]. Recent high quality data reported by Wang et al. involving the multiplex profiling of peritoneal metastases from gastric adenocarcinoma showed that the mesenchymal-like molecular subtype had a discriminating response to chemotherapy (31% vs 71%) [52]. 

Patients with positive peritoneal spread but without radiologically measurable diseases have traditionally been excluded in non-adjuvant clinical trials as RECIST measurements would not be possible. The question of the effectiveness of palliative chemotherapy in this subgroup is hence debatable, as they have been inadequately studied. As the biology of gastric cancers are better understood, this is likely going to change. One example is the ongoing Glow study (a randomized Phase 3 study of Zolbetuximab + CAPOX compared to placebo + CAPOX as first line treatment of patients with CLD18.2-positive, HER2-negative metastatic gastric cancer). CLD 18.2 is enriched in diffuse gastric cancers and these patients often do not have radiologically measurable disease. Progression-free survival is the primary endpoint for this study and, patients without radiologically-measurable diseases have been allowed to enroll to ensure adequate accrual.

## 4. Liquid Biopsy as a Possible Solution for Clinical Monitoring

Disease response assessment and therapeutic monitoring is difficult for radiologically-occult diseases, and there is a need for more objective measurements. The liquid biopsy is an emerging noninvasive diagnostic approach involving the isolation and analysis of circulating tumor markers via the peripheral venous sampling of a small volume of blood; this approach has the potential to fulfill this unmet need. It can serve as an adjunct to radiology, conventional tumor markers, or tissue biopsy in the diagnosis or surveillance of gastric cancer patients.

Data are still emerging, but available literature appears to validate a role for liquid biopsy in gastric cancer patients. The most promising potential markers are DNA fragments of cell-free DNA (cfDNA), which are called circulating tumor DNA (ctDNA), and circulating tumor cells (CTCs), but the role of circulating microRNA, tumor-educated platelets, tumor-derived exosomes, and non-coding RNAs (ncRNA), have been studied as well [53]. A description of various liquid biopsy platforms, advantages, disadvantages, and current statuses in clinical adoption is summarized in Table 3. A summary of the available literature on the use of liquid biopsy and potential biomarkers for the evaluation of therapeutic efficacy in GC is presented in Table 4.

### 4.1. Circulating Tumor Cells (CTCs)

CTCs are rare cells that are shed into the circulation of malignant tumors. They are found at low concentrations and have very short half-lives. The CTC technique is the oldest liquid biopsy platform, and the CellSearch^®^ (Veridex LLC, Raritan, NJ, USA) immunomagnetic system is FDA-approved for identifying CTCs in metastatic breast, colorectal, and prostate cancers. This technique uses antibodies against epithelial markers CK8/18/19 and EpCAM, as well as CD45, to differentiate from circulating peripheral White Blood Cells [57]. It has also been commonly used in the identification of CTCs in gastric cancer. CTCs have been shown to have high specificity for GC, though their sensitivity is limited (99% and 42%, respectively) [71]. There is now increasing evidence that suggests that CTCs can be useful in disease monitoring in advanced gastric cancer [56,57,62,63]. The yield of CTCs appears to be discriminatory with regards to histology, with intestinal adenocarcinoma being associated with higher levels of CTCs compared to signet-ring cells [72]. Tumors undergoing epithelial-to-mesenchymal transition (EMT) that would be enriched in diffuse/signet rig gastric cancers lose epithelial markers while mesenchymal markers are elevated. An EpCAM-mediated CTC detection technique would hence be less reliable in these patients. Vimentin and wwist have been studied as potential markers for those CTCs and circulating tumor microemboli (CMT) that are undergoing the epithelial-to-mesenchymal transition (EMT) with encouraging results [62,73].

### 4.2. Cell-Free DNA (cfDNA)/Circulating Tumor DNA (ctDNA)

Cell-free DNA, or circulating tumor DNA (cfDNA or ctDNA), is free-floating DNA in the plasma that is derived from cancer cells and reliably demonstrates the genetic makeup of the primary tumor. Additionally, ctDNA has the potential to provide an insight into treatment resistance patterns, especially with regard to targeted therapy [74]. While limited research has been conducted regarding ctDNA in gastric cancer patients, ctDNA levels have been shown to be reliable markers of disease status and prognosis in cancer patients postoperatively. A recently published article illustrated how significantly ctDNA can impact clinical practice. In this seminal article, samples from patients in the CRITICS trial (a phase III randomized controlled study of perioperative treatment in patients with operable gastric cancer) were analyzed for ctDNA after filtering out alterations from matched white blood cells. The presence of ctDNA predicted recurrence when analyzed within nine weeks after preoperative treatment. After a median follow-up of 42 months, all 11 patients without detectable tumor-specific mutations at the postoperative timepoint were alive and free of recurrence. On the other hand, six out of nine patients with detectable tumor-specific mutations at the postoperative timepoint developed disease recurrence and died from metastatic diseases [75]. Another recent paper in patients with operable colon cancer demonstrated a similar utility of cDNA. In this study, patients with detectable ctDNA after the completion of adjuvant chemotherapy were shown to have relapse free survival of just 30% at three years compared to 77% when ctDNA was undetectable (HR, 6.8; 95% CI, 11.0–157.0; *p* < 0.001) [76]. Many of the same molecular targets for gastric CTCs have been researched for ctDNA in gastric cancers including TP53 [54] and HER2 [55], and increasing levels have been associated with a poorer prognosis [77]. One recent study reported that the molecular tumor burden index (mTBI) was predictive of treatment response with a sensitivity of 94% [74]. The clonal temporal evolution of ctDNA in individual patients has a potential to explain drug resistance to targeted therapy [72,78]. Various isolation and analysis methods are being developed for ctDNA detection including next-generation sequencing, real-time PCR, digital droplet PCR, Scorpion ARMS and PNA-LNA PCR.

### 4.3. Circulating mRNA

Circulating mRNA, like ctDNA, has also shown promise in providing a non-invasive approach to detecting tumor genetic alterations [79]. CXCR is of particular interest in this patient population, as CXCR expression has been significantly associated with peritoneal metastasis [80]. Additionally, many different genes and their respective circulating levels of mRNA have shown significant associations with chemosensitivity to certain regimens in gastric cancer patients [61,65,79,81,82,83]. However, limited data exist for dynamic monitoring of mRNA levels in gastric cancer patients.

### 4.4. Tumor-Educated Platelets (TEP)

Tumor-educated platelets (TEP) are an emerging technology and a powerful tool that leverage the tumor RNA that is contained in the host platelets. Unlike other liquid biopsy platforms, they are highly stable [84] and hence have immense potential for dynamic monitoring of cancers. They have been studied in various tumor types, but data with gastric cancer are currently lacking.

### 4.5. Circulating Long-Noncoding RNA (Lnc-RNA)

Long-noncoding RNAs (Lnc-RNA) have also begun to be investigated in gastric cancer. While dynamic monitoring studies are limited, a few studies have shown that the serum levels of a variety of Lnc-RNA respond to treatment, including highly upregulated in liver cancer (HULC) [70] and H19 [69]. Given the stability of Lnc-RNAs in serum relative to other nucleic acids, these represent a potential future direction for research.

## 5. Conclusions

Overall, liquid biopsy is a promising avenue for noninvasive diagnostic and therapeutic monitoring, which requires further study, specifically with prospective trials. Liquid biopsy is a low risk, noninvasive procedure; the only risk involved is the risk of venipuncture. It is less resource-intense at sites of clinical care delivery (hospitals and oncology clinics) and does not require a specialty expertise for acquisition. Blood is shipped to regional processing centers and can then be regulated to have the highest desired quality for optimal clinical care. Liquid biopsy can potentially obliterate the subjective biases that exist in the radiological monitoring of the peritoneal disease burden. With mass adoption, cost is likely to come down substantially in a competitive market and may pave the way for cost-effective serial monitoring. Several potential markers exist for gastric cancer specifically, and methodologies that combine these biomarkers into single assays should be investigated. This will allow for gastric cancer patients with radiologically-occult metastatic diseases to be monitored more effectively.

## Figures and Tables

**Table 1:** The Cancer Genome Atlas (TCGA) Classification.

Epstein-Barr Virus- Positive (EBV, 9% of Cases)	Microsatellite Instability (MSI, 22% of Cases)	Genomically Stable (GS, 20% of Cases)	Chromosomal Instability (CIN, 50% of Cases)
- Characterized by EBV positivity. - Mostly located in the gastric fundus or body (62%). - Higher prevalence of DNA promoter hypermethylation (including CDKN2A promoter hypermethylation in all tumors). - Frequent PIK3CA (80%), ARID1A (55%) and BCOR (23%) mutations. - Has higher prevalence of DNA hypermethylation than any cancers reported by TCGA. - Amplification of 9p24.1 locus containing genes encoding JAK2, PD-L1 and PD-L2 has been seen in 15% of the tumors.	- Characterized by hypermutated genome, DNA hypermethylation and MLH1 silencing. - Occurs in relatively older ages (median 72 years). - Mutations in PIK3CA (42%) have frequently been seen.	- High percentage of invasive phenotype (73% had diffuse histology). - Low somatic copy-number aberrations - CDH1 somatic mutations are enriched in this subtype (37%). - Newly discovered RHOA mutations and CLDN18-ARHGAP rearrangements have been seen in 30% of cases and these two mutations have been found to be mutually exclusive. - Both RHOA mutations and CLDN18-ARHGAP rearrangements (by affecting RHOA proteins) are thought to result in disparate growth patterns and a lack of cellular cohesion that are hallmarks of diffuse tumors.	- Characterized by high somatic copy-number aberrations and frequently have intestinal histology. - TP53 mutations have been seen in 73% of cases. - Amplifications of genes in the receptor tyrosine kinase–Ras pathway, including VEGFA, EGFR (10%), ERBB2 (24%), ERBB3 (8%), FGFR2(8%) and c-Met (8%), and amplifications of cell cycle mediators (CCNE1, CCND1 and CDK6 have been frequently seen.

**Table 2:** Asian Cancer Research Group (ACRG) classification.

MSI-High (MSI, 22.7% of Cases)	Microsatellite Stable/Epithelial–Mesenchymal Transition (MSS/EMT, 15.3% of Cases)	Microsatellite Stable/TP53 Intact (MSS/TP53^+^, 26.3% of Cases)	Microsatellite Stable/TP53 Loss (MSS/TP53^−^, 35.7% of Cases)
Best prognosis More than half of the cases diagnosed have been in early stages (I/II). This subset is similar to the MSI TCGA subset.	More frequent in the gastric antrum (37%) and body (45.6%). Majority with diffuse-type histology (80.4%) and advanced stages (III/IV—80.4%). Worst overall prognosis and a higher chance of recurrence. The patients in this group have been found to be 10 years younger than the other group (median age 53).	EBV infection occurs predominantly in this subgroup. This subtype has the second-best prognosis.	Less favorable prognosis compared to MSI and MSS/epithelial/TP53^+^. Better prognosis than MSS/EMT. Highest rate of TP53 mutations (60%) and TCGA. CIN tumors are enriched in this subgroup.

**Table 3:** Biomarker of liquid biopsies studied in gastric cancers.

Biomarker	Description	Advantage	Disadvantage	Clinical Adoption
CTCs	Rare cells that are shed from tumors into circulation.	High specificity. Data available on treatment response monitoring.	Low sensitivity. Short half-life.	Potentially applicable but not FDA approved in gastric cancer. CellSearch analysis of CTCs is FDA approved for prognostication in metastatic breast cancer, colorectal cancer, and prostate cancer
cfDNA	Freely circulating single- or double-stranded DNA, shed by either living or dying tumor cells.	Promising platform with well-developed analysis techniques like PCR and NGS. Can inform about mutations in therapeutic targets responsible for drug resistance. Coupled with AI, this platform has the potential to be reliable.	Short half-life (minutes to hours).	ctDNA detection of Her 2 neu is possible through multiple vendors: FoundationOne, Tempus and Gaurdant, but not widely used.
microRNA	Short, stable, noncoding RNA gene products made up of 19–25 nucleotides.	Relatively easily isolated in plasma. Stable over time if properly acquired. Potentially detectable in other body fluids—saliva, urine, etc.	Early in development. Lack of consistent data.	Not commercially available and not clinically used.
Tumor-educated platelets	Tumor cells interact with platelets by activating surface receptors, which alter the expression of platelet cytokines and mRNA.	Highly stable, abundant, and reliable.	Little information in gastric cancer and early in development.	Not commercially available and not clinically used.

**Table 4:** Liquid biopsy for therapeutic monitoring in gastric cancer patients.

Biomarker	Description	Method	Sample	Clinical Significance
TP53 [54]	ctDNA	qRT-PCR	42 AGC	Circulating levels decrease post-gastrectomy and increased levels associated with disease recurrence.
HER2 [55]	ctDNA	ddPCR	60 GC	Plasma HER2 amplification decreased post-gastrectomy, increased levels reliably associated with recurrence.
EpCAM, CK8, CK18, CK19, and CD45- [56]	CTCs	CellSearch^®^	52 GC	CTC levels post-therapy significantly associated with clinical response.
EpCAM, CK8, CK18, CK19, and CD45- [57]	CTCs	CellSearch^®^	138 AGC	Decreased CTC levels post-chemotherapy significantly associated with therapeutic response.
EpCAM, CK8, CK18, CK19, and CD45- [58]	CTCs	CellSearch^®^	251 GC	Patients with detectable CTCs post-therapy significantly more likely to have recurrence (75% of patients with CTCs post-therapy).
EpCAM, CK8, CK18, CK19, and CD45- [59]	CTCs	CellSearch^®^	136 AGC	Circulating CTCs more common in peritoneal metastasis than liver, circulating levels associated with response to therapy.
EpCAM, CK8, CK18, CK19, and CD45- [60]	CTCs	CellSearch^®^	130 GC	Change in serum levels of CTCs correlated with stage and treatment effect.
CEA, CK19, hTERT, MUC1 [61]	CTCs	High-throughput colorimetric membrane array	64 GC	Higher serum levels associated with advanced stages and recurrence/metastasis.
Vimentin, Twist, CK8, CK18, CK19, CD45- [62]	CTCs	CanPatrol^®^ (RNA-ISH)	44 GC	Decreased circulating CTCs and decreased mesenchymal-like features in response to treatment.
Chromosomes 7 and 8 [63]	CTCs	FISH	80 AGC	CTCs significantly decreased after neoadjuvant therapy with DOF +/− bevacizumab and subsequent gastrectomy.
Chromosome 8 [64]	CTCs	SE-iFISH	31 AGC	Comparison of chromosome aneuploidy in CTCs before and after treatment significant for treatment response.
Bmi-1 [65]	mRNA	RT-PCR	89 GC	Circulating levels significantly decreased postoperatively.
CXC Receptor 4 (CXCR4) [65]	mRNA	RT-PCR	89 GC	Circulating levels significantly decreased postoperatively; higher levels significantly associated with peritoneal metastasis.
miR-17-5p [66]	miRNA	qRT-PCR	14 GC	Circulating levels significantly reduced postoperatively and significant increase seen in relapse.
miR-18a [67]	miRNA	qRT-PCR	104 GC	Circulating levels significantly decreased postoperatively.
miR-20a [66]	miRNA	qRT-PCR	14 GC	Circulating levels significantly decreased postoperatively.
miR-196a/b [68]	miRNA	qRT-PCR	23 GC	Circulating levels significantly decreased post-operatively.
H19 [69]	Lnc-RNA	qRT-PCR	43 GC	H19 levels significantly decreased post-gastrectomy, however no significant difference between levels in healthy controls and GC patients.
HULC [70]	Lnc-RNA	qRT-PCR	173 GC	Circulating levels significantly decreased postoperatively.

AGC: advanced gastric cancer; CTCs: circulating tumor cells; ctDNA: circulating tumor DNA; ddPCR: digital droplet PCR; FISH: fluorescent in situ hybridization; GC: gastric cancer; Lnc-RNA: circulating long non-coding RNA; miRNA: circulating microRNAs; mRNA: circulating messenger RNA; qRT-PCR: quantitative real-time PCR; SE-iFISH: subtraction enrichment and immunostaining-fluorescence in situ hybridization.
